# Healthcare professionals’ perceptions on providing support to informal carers within stroke care

**DOI:** 10.1371/journal.pone.0311915

**Published:** 2024-10-15

**Authors:** Melissa Jammal, Gregory S. Kolt, Karen P. Y. Liu, Nariman Dennaoui, Emma S. George

**Affiliations:** 1 School of Health Sciences, Western Sydney University, Sydney, NSW, Australia; 2 Department of Rehabilitation Sciences, The Hong Kong Polytechnic University, Hong Kong SAR, China; 3 Translational Health Research Institute, Western Sydney University, Sydney, NSW, Australia; Spreeha Foundation Bangladesh / The University of Sydney / The University of Southern Queensland, AUSTRALIA

## Abstract

**Background:**

The sudden nature of stroke onset does not provide carers with sufficient time to prepare for the demands associated with caring. Healthcare professionals can have a vital role in providing carers with support and training, which may reduce carer stress and strain, and allow for better health and rehabilitation outcomes for the stroke survivor. The experiences of healthcare professionals on supporting carers in stroke care, however, remain unclear.

**Objective:**

To understand the experiences and perceptions of healthcare professionals working in stroke care on implementing resources and support to informal stroke carers.

**Methods:**

Semi-structured interviews were conducted with 11 healthcare professionals (5 occupational therapists, 5 physiotherapists, 1 psychologist) with at least 12 months’ experience in working with stroke survivors. Interviews ranged from 25–70 minutes in duration, and were recorded, transcribed, and analysed using thematic analysis.

**Results:**

Three overarching categories that were explored were: (1) Experiences of working in stroke care and supporting carers; (2) Recommendations for a program designed for carers; and (3) Future priorities in stroke care. Participants discussed a variety of strategies they utilised to support carers including collaborating with other healthcare professionals and utilising skills and experience. Healthcare professionals highlighted the need for additional resources that are designed specifically for carers and explored key topics including carer stress and fatigue, support services, stroke education, and how to look after oneself. Participants identified priorities for stroke care including additional professional training and resource availability.

**Conclusion:**

This study provided a unique insight from the perspectives of healthcare professionals on supporting carers. Participants identified the need for additional training and resources to equip healthcare professionals to better support carers. Future programs designed for carers should be informed by the needs and experiences of both informal carers and healthcare professionals.

## Introduction

Globally, stroke is the second leading cause of death and third leading cause of death and disability combined [1, 2]. Following a stroke, informal carers play a crucial role in assisting stroke survivors with their daily activities, offering emotional support, and with their rehabilitation [3, 4]. An informal carer is an individual who provides unpaid support to an individual with a disability or condition within the context of an existing relationship such as a family member or friend [5]. Due to the unique and abrupt nature of stroke onset and often short length of stay in hospital, it can be challenging for stroke survivors and their carers as they need to quickly adapt and respond to the change in health status and support needs [6–9].

Healthcare professionals are well positioned to play a vital role in supporting not only stroke survivors, but also their carers through providing training, support, and education [10]. A study by Lilley et al. [11] found that carers who received ongoing support and information from healthcare professionals reported a feeling of reassurance. Carers often rely on healthcare professionals as their main source of information [12]. It is evident, however, that healthcare professionals can experience healthcare delivery issues that impact on the provision of support to carers. These issues include a focus on patient-centred care as opposed to family-centred care, and the inability for carers to access such support during the usual work hours of hospital-based healthcare professionals [10]. As a result, following hospital discharge of the stroke survivor, many carers report feeling uncertain about the future and underprepared to support the stroke survivors’ transition home [13–15]. Consequently, carers can be at risk for experiencing carer stress, strain, and burden leading to poorer health outcomes for themselves and reduced quality of care for the stroke survivor [15, 16].

To date, little research has examined the perceptions and experiences of healthcare professionals within stroke care on supporting carers. A study by Cameron et al. [10] explored the support needs of carers over time from the perspective of both stroke carers and healthcare professionals. This study identified a need for change in stroke care delivery to better support the changing needs of carers across the care continuum. Further, a study by O’Brien et al. [17] found that healthcare professionals recognise the challenges and needs of stroke carers and highlighted the importance of incorporating the experiences of healthcare professionals to inform the development of programs specifically for carers. With only minimal published research in this area, a clear need exists to explore the experiences of healthcare professionals to inform recommendations for practice and future support programs.

To address carer needs, previous programs designed for stroke carers have typically been delivered by healthcare professionals and included at least one of three components: education about stroke and available resources, emotional support (i.e., counselling, peer support), and skills training (i.e., problem solving, stress management) [18]. Several systematic reviews, however, have concluded that there is a lack of high-quality intervention studies and therefore, there is still a need to confirm efficacy of carer interventions [6, 18].

It is well established that insights from participants’ experiences, perspectives, and needs are essential to inform the development of relevant client-centred interventions and services [19, 20]. Despite this, only a few intervention studies have utilised qualitative methodologies to explore perceptions of relevant individuals or to conduct formative work when designing programs [21]. Therefore, it is important to understand the experiences of healthcare professionals who deliver health care services to stroke survivors and carers to improve clinical practice and inform program development.

The aim of our study was to address the current gaps in the literature by examining (through qualitative methodology) the experiences and perceptions of health professionals working in stroke care on how to best support carers, and to identify what is feasible to deliver within a clinical setting. The findings from this study will be utilised to inform the development of a support program to promote the health and wellbeing of informal carers. We aimed to answer the following research questions:

1. What are the experiences of healthcare professionals working in stroke care on implementing resources and support to informal carers in their clinical practice?
2. What recommendations can be made to inform the development of an accessible and acceptable carer-designed support program?
3. What clinical-based recommendations can be made to improve support for carers within stroke care?

## Methods

This study was reported in accordance with the Consolidated Criteria for Reporting Qualitative Studies (COREQ) [22]. A qualitative descriptive study design was used within this study. This approach is a method of naturalistic inquiry whereby researchers seek a precise account of participants’ experiences and events [23]. The qualitative descriptive approach provides a method to understand important clinical issues and contribute to change and improvement in practice settings [24]. This approach was chosen to understand the complexities involved in supporting stroke carers in practice and to help inform recommendations for practice.

### Study design

Between February 2024 and April 2024, university-qualified and registered healthcare professionals from Australia were purposively sampled through distribution of flyers via: (1) relevant closed social media groups (i.e., Facebook, LinkedIn); (2) private allied health clinics; and (3) a stroke education website platform. By using a purposive sampling strategy, recruitment targeted healthcare professionals with specific experience in providing services to stroke survivors and their carers. Healthcare professionals were asked to scan a QR code or click on a link to complete an eligibility survey via Qualtrics. To be eligible for this study healthcare professionals had to:

- Be employed in Australia.
- Have at least 12 months’ experience working directly with stroke survivors and their carers.
- Be a qualified healthcare professional registered with their profession’s National Board in Australia.

A total of 17 healthcare professionals completed the survey and met the eligibility criteria. These healthcare professionals were contacted via email or telephone by a member of the research team and asked to indicate their availability for an individual interview. Following this, thirteen participants agreed to participate. Participants were then asked to complete an online questionnaire to collect basic demographic data (i.e., age, sex, education, employment status). Two healthcare professionals who initially completed the initial survey subsequently declined to participate in an interview due to travel or personal reasons. Participants were recruited until information power was reached [25]. Once written informed consent was obtained, participants were asked to indicate a convenient time and place for an interview.

### Data collection

Semi-structured interviews were conducted either online or in person in a private room at a local library selected by participants. A semi-structured interview topic guide was used to guide conversation and prompts (see S1 Appendix). The topic guide was developed by the authors and then pilot tested with three healthcare professionals and adjusted accordingly to ensure suitability of questions. Prior to commencement of the interview, oral consent was obtained, and participants were advised they can withdraw from the study at any point. One author (MJ) facilitated the interviews through using the topic guide to ask questions and provide prompts, whereas another author (ND) was present to take detailed notes and capture key points and other important observations such as body language. Participants were asked to reflect on their experiences in supporting carers within stroke care and to suggest recommendations for improving practice. All interviews were audio-recorded and transcribed verbatim for analysis. Each participant was assigned a code and any identifying information was removed from the transcripts to maintain confidentiality. Participants were provided a $20 AUD gift card for their participation.

### Data analysis

Audio recordings were professionally transcribed and checked by the lead researcher (MJ). Transcripts were imported into NVivo and analysed using the six phases of reflexive thematic analysis outlined by Braun and Clarke [26], where data were collated, examined, coded, reviewed, defined, and generated into themes. Thematic analysis is a data-driven method for identifying, analysing, interpreting, and reporting patterns and themes within a dataset [26]. A hybrid approach comprising a combination of inductive and deductive analysis was used to harness the advantage of both methods [27]. An inductive approach was used to provide a flexible and adaptable approach that allowed for the identification of key themes and patterns based on the experiences of participants [26], whereas a deductive process was used to provide a structured analysis of data through the use of framework to organise codes into three overarching categories (study aims) [27].

Initially, one author (MJ) systematically read through all transcripts to familiarise themselves with the data and used annotations to make initial notes. Following this, other authors systematically read through the transcripts, highlighting relevant segments, and tagging these segments with initial codes. Codes with similar meaning were then grouped together into initial themes and then were reviewed, revised, and refined until organised into a set of themes within each overarching category. The authors were able to reach a consensus concerning themes and subthemes through a process of ongoing discussion. Analysis continued until data adequacy was achieved which was determined by the richness of data supporting themes [28].

#### Rigour and trustworthiness

The lead investigator (MJ), a female PhD candidate and qualified and registered occupational therapist with experience working in stroke care, facilitated all interviews. To be aware of biases, assumptions, and positioning, the lead investigator engaged in an ongoing process of reflection through the use of a reflexive journal [29], allowing them to reflect on their positioning and life experiences related to supporting carers in stroke care as an occupational therapist. The researcher was also able to reflect on their own role in supporting carers in clinical practice and their role as an informal carer. The lead author (MJ) also kept detailed notes of their decisions, actions, and thoughts throughout data collection and analysis [30]. This practice aimed to encourage reflexive consideration enabling the researcher to acknowledge and address potential sources of bias [31]. Further, member checking was employed by providing participants with an opportunity to read through transcripts and deliver any comments and/or corrections [32]. Minor adjustments were made by two participants to eliminate information around the specific healthcare setting in the transcripts. These various techniques were used to enhance trustworthiness and credibility of data analysis.

### Ethical considerations

This study was approved by the Western Sydney University Human Research Ethics Committee (approval number H15582). Written informed consent and verbal consent was obtained from participants prior to the commencement of the interviews.

## Results

Table 1 presents the sociodemographic information for participants. A total of 11 healthcare professionals participated in individual interviews, including 5 occupational therapists, 5 physiotherapists, and 1 psychologist. The clinical experience of healthcare professionals ranged from 3 to 23 years. Participants were from four states within Australia (New South Wales, Queensland, South Australia, and Victoria). Interviews ranged from approximately 25 to 71 minutes in duration.

**Table 1:** Sociodemographic characteristics of healthcare professionals.

Participant	Age	Gender	Profession	Clinical experience	Clinical Setting
1	29	Female	Occupational Therapist	8 years	Acute neurology ward
2	34	Male	Physiotherapist	12 years	Stroke rehabilitation ward
3	31	Female	Physiotherapist	10 years	Acute neurology ward
4	29	Male	Occupational Therapist	8 years	Private inpatient rehabilitation
5	26	Female	Physiotherapist	3 years	Acute and neurological rehabilitation
6	25	Female	Occupational Therapist	3 years	Community (National Disability Insurance Scheme)
7	33	Female	Occupational Therapist	10 years	Inpatient neurological rehabilitation
8	41	Female	Physiotherapist	16 years	Inpatient rehabilitation
9	39	Female	Occupational Therapist	14 years	Community (National Disability Insurance Scheme), private
10	25	Female	Physiotherapist	3 years	Inpatient rehabilitation
11	61	Female	Psychologist	23 years	Community (National Disability Insurance Scheme), private

The 3 overarching categories that were used were: (1) Experiences of working in stroke care and supporting carers (3 themes, 4 subthemes); (2) Recommendations for a program designed for carers (2 themes); and (3) Future priorities in stroke care (2 themes) (See S2 Appendix). All 3 categories are interrelated and reflect the experiences of healthcare professionals and the complexity of supporting informal carers within practice. Table 2 lists the three categories, main themes, and subthemes.

**Table 2:** Categories, main themes, and subthemes.

Category	Themes	Subthemes
Experiences of working in stroke care and supporting carers	How healthcare professionals support carers	Key strategies from the field Collaboration between healthcare professionals
	Skills and knowledge of healthcare professionals	
	Clinical practice: Priorities and pressures	Challenges in the workplace Priorities in practice
Recommendations for a program designed for carers	Content and information delivery	
	Timing of support	
Future priorities in stroke care	Empowering health care professionals	
	Less judgement, more structure	

### Experiences of working in stroke care and supporting carers

Participants discussed their experiences of working in stroke care and supporting carers in practice. Three themes were identified to capture these experiences: (1) How healthcare professionals support carers; (2) Skills and knowledge of healthcare professionals; and (3) Clinical practice: priorities and pressures.

#### How healthcare professionals support carers

Participants discussed several ways they support carers within stroke care, and these are classified into 2 subthemes: (1) Key strategies from the field; and (2) Collaboration between healthcare professionals.

*Key strategies from the field*. Participants discussed various strategies they utilised to support informal carers, including supervision of junior staff by a senior clinician, employing a “key worker” approach, and utilising a trial of care period. The supervision of junior clinicians was identified by participants as an important strategy to ensure healthcare professionals were supported with the decision-making process. For example, one participant reported:

*“*When it is a meeting that we have around could they be a carer or couldn’t they, we have a local practice where a more senior staff will go with the new rotational member to that meeting to help with decision making*”* (Participant 8).

A common view among participants was the importance of ensuring there is a “key worker” who is allocated to each stroke patient. Participants reported that the “key worker” will often be the health professional that will spend the most time with the patient or has established the greatest rapport. Other participants discussed the vital role of a key worker in being a primary point of contact for carers and patients as well as communicating important information. One participant reflected on the importance of the key worker in the statement: “We will have a key worker that communicated with the family so it’s not all being bombarded from different professions” (Participant 7).

Another strategy utilised by healthcare professionals was described as a “trial of care” or weekend leave. Participants discussed the importance of allowing carers to trial providing care for a 24 to 48-hour period to determine if they experienced any difficulties. The use of a trial of care was also seen as an important strategy for carer training or assessment. One participant reported the use of an independent living unit (ILU) where carers could stay overnight with the stroke survivor in a separate unit or area.

Another participant reflected on the importance of using the ILU to ensure the carer can trial providing care. This was seen as important to allow the healthcare professional to make any adjustments if difficulties arose during the ILU stay.

Many healthcare professionals identified the importance of using these strategies to provide carers with an opportunity to identify the extent of their involvement in supporting the stroke survivor. This was discussed as encouraging shared decision making.

“You really don’t know how that person [carer] is going to go until you’ve actually trialled it, and we say to carers as well, you might start being the carer and you might decide that it’s actually not for you*”* (Participant 8).

“Having those sessions where I am teaching them [carer] and liaising with them to see if that is something that they would be comfortable doing moving forward and taking the patient home… practicing that over and over again to make sure it is a shared decision*”* (Participant 5).

Some participants discussed the use of trial of care and ILU to ensure the carer felt confident in their ability to provide care and provide additional opportunities for practice of carer skills such as transfers. Other participants discussed the importance of weekend leave or a trial of care to ensure the carer would be adequately prepared and aware of the demands of caring.

“We certainly make sure to put a weekend leave in as well because often that overnight can be quite different for carers as well and having been visiting in the hospital during the day, things might look different overnight. They realise the task is a lot bigger than what they expected” (Participant 8).

*Collaboration between healthcare professionals*. Participants discussed the importance of collaborating with other healthcare professionals to ensure there was a shared responsibility for supporting carers. Collaboration was discussed as a key strategy that led to better outcomes for carers due to being seen as a more holistic approach.

Participants identified a variety of ways they collaborate with other healthcare professionals including attending daily meetings, family meetings, through “corridor discussions”, and joint sessions. Participants reported these meetings were crucial as they provided an opportunity for the healthcare team to create a plan, discuss any barriers, raise concerns, and discuss strategies to overcome identified barriers.

“It really helps that we’re all on the same ward in the same environment. So we have a lot of those kinds of corridor conversations. We just casually walk past each other go, oh, have you seen this patient? How did they go with you? Are there any kind of strategies I need to think about when I go in and see them?” (Participant 3).

One participant identified the importance of collaboration with other professionals to determine if they have identified similar concerns or barriers. Other participants reflected on how meetings often provide an opportunity to discuss concerns in the home environment which then allowed the team to develop a plan to overcome such concerns. Participants also discussed the importance of collaboration to share important information and contribute different strengths to the supporting the carer practically and emotionally.

“We all just bring our different strengths. I definitely would be the go-to from a practical point of view and our psych and our social worker as well who’s got a lot of experience, definitely more around that coping adjustment” (Participant 8).

The use of joint sessions was discussed as another way that healthcare professionals could collaborate to provide holistic care and education to the carer. Participants reported the importance of joint sessions to ensure health professionals were developing strategies that could reduce the load on carers. For example, one participant reflected on a situation that required the use of joint sessions, “as a team we came up with a strategy, like a communication book for homework… we wanted his [stroke survivor’s] wife to take a step back from being the case manager of all the support workers” (Participant 6).

For one participant, joint sessions were used to specifically check in on the health and wellbeing of the carer.

“Some of the little sneaky things we do if we’re doing a home visit is when we’ve got two staff members on the visit, we always have one staff member do something with the patient… and the other one [staff member] is in the background sneakily having a chat with the carer and just checking in with them… because often that person is not going to be honest in front of the person they’re caring for” (Participant 8).

For other participants joint sessions provided a convenient method where the healthcare professionals could collect information and complete assessments together. One participant discussed that this process allowed for healthcare professionals to “share the load”. This collaboration was seen as pivotal to ensuring a more “holistic view”.

#### Skills and knowledge of healthcare professionals

Participants discussed the importance of their clinical experience, training, and knowledge which was seen as pivotal to supporting the carer. Many participants reflected on how their clinical experience provided them with the confidence and knowledge of when to engage the carer, different ways to provide education, and how to identify signs of carer burnout. For example, one participant advised, “having been an OT [Occupational Therapist] now for about nearly 15 years, I feel that I have a better idea about when to involve the carers, and when not to stress the carers” (Participant 9).

One participant reflected on their experience working with stroke survivors in both a hospital inpatient setting and in a community setting, allowing them to more deeply observe carer needs and risk factors for carer burnout.

“At this stage in my career I am quite confident, especially having worked in community and seen people a few months down the track post discharge and then worked in inpatient as well. So I do have a bit more of an understanding about what is realistic and what the risk factors for burnout for those carers” (Participant 7).

Other participants discussed their lack of specific training and knowledge of carer services and resources available, which impacted on their confidence when supporting carers. Many participants discussed feeling confident in providing practical support for carers such as education and training, however, reported lacking confidence in providing emotional support to the carer.

“When providing education on symptoms related to function… I am quite confident, but that’s probably where my confidence with it ends–In terms of providing any sort of psychosocial support to them [carer], and their carer role that they’re taking on, no, I don’t have a lot of confidence” (Participant 1).

Many participants reported they did not receive any formal training on supporting carers and they were unable to locate any courses to assist in this regard. These participants discussed that professional development courses were often focused on the person who has had a stroke rather than the carer. Participants reflected on the lack of training which often resulted in them having to “learn on the job”.

“Within the OT [Occupational Therapy] department at work we do have PD [professional development] that’s offered but it is very broad in general so it’s not always targeted to these specific areas … there is stroke PD training it’s often you know upper limb therapy or cognitive retraining, there is not necessarily that much out there in relation to actual carer training and what’s required on discharge. So it’s a lot of learn on the job” (Participant 7).

#### Clinical practice: Priorities and pressures

Participants discussed how within clinical practice there were priorities and pressures placed on healthcare professionals that impacted on their ability to support carers. Within this theme, 2 further subthemes were identified: (1) Challenges in the workplace; and (2) Priorities in practice.

*Challenges in the workplace*. Participants discussed several workplace-related external pressures and challenges that impacted on their ability to provide support to carers. These pressures included staffing issues, resource availability, working hours, language barriers, bed pressure, billables, and funding bodies.

One of the major challenges identified by participants was staffing issues within their workplace. These staffing issues included a lack of staff, high turnover rate of staff, difficulty recruiting healthcare professionals, and use of long service leave and sick leave. Participants reflected on how these staffing issues meant there were not enough staff to provide patient care and support carers.

“We know that we can’t deliver as much therapy as we’d like just because of the staffing issues. So number one reason why we’re not just going around talking to people and calling people when we need to would be this” (Participant 2).

Another challenge identified by participants was the limited availability of resources designed for carers. The majority of participants reported often providing carers with “generic handouts” from the internet or downloading resources from stroke organisations. A few participants discussed how the resources available often were not designed for carers which meant that these resources were not beneficial to carers or were not used.

“Well lack of resources as well probably, like I said, it’s literally just if I want to provide education to the carer, I’ll jump on the Stroke Foundation or stroke pathway websites… and I’ll print them out and just give them a bit of an information bomb, stuff that’s probably just going to go in the top drawer and not be looked at” (Participant 4).

Other participants reported they were aware of the lack of resources available for carers, however, they often did not have the time or staffing to create additional resources that were designed specifically for carers.

“But one of them [priorities] would be like that kind of ready to go resource like we, it’s something that’s on our list of something that we want to do. But as there is a lot of things and it’s the time to actually put something like that together and having something that can be flexible” (Participant 10).

The usual work hours of 8:00am to 4:30pm within a hospital setting were identified as another barrier to supporting carers. Participants discussed how their work hours would often result in the carer not being available. Some participants reported having difficulty contacting the carer to organise training or support. One participant reflected on the strict hospital visiting hours which also impacted on the carer’s availability. Other participants discussed how carers are often busy with other commitments and unable to discuss the patient until after hours.

“They [carers] are very busy as well. So trying to get a hold of those people and connect with them it’s often after hours it’s often when you know school goes out or whatever four o’clock in the afternoon where they actually have time” (Participant 8).

One participant reflected on the difficulty of engaging carers from a culturally and linguistically diverse background where English is their second language.

“We [healthcare professional] might not have the Arabic or the Vietnamese or whatever it is to try to educate and convince them [carer]. So there is loads of barriers to try to get them to believe us” (Participant 2).

Other participants discussed the difficulties of meeting the expectations of carers with a low health literacy, and who may not understand the role of the healthcare professionals.

“Potentially low health literacy might potentially be a bit of a barrier as well, in regards to sometimes people have very different expectations of what kind of services and supports the hospital can give. They expect you know, 24/7 care, that people that will come in and do all this extra stuff and 24/7 therapy and the kind of thing that I’d love to be able to give, we just unfortunately can’t” (Participant 3).

A few participants reported experiencing challenges with engaging carers due to a mistrust in the medical system or of healthcare professionals. This meant that healthcare professionals had to spend additional time building rapport with stroke patients and their carers.

“I think a lot of the time you know they’ve had it’s a horrible experience and I think that’s where a lot of the mistrust comes from…I feel like a lot of the time, by the end of their admissions we often have a really good rapport but we are are spending so much time getting to that point” (Participant 7).

Another challenge reported by participants was the pressure to discharge patients within a timely manner. Many participants discussed the difficulty in trying to support and prepare carers for their role within a short timeframe. This was identified as particularly difficult for participants who worked in an acute or rehabilitation setting. Participants reflected on how the pressure from health services to discharge patients meant that carers would often have to provide additional support for patients that had high care needs.

“I think there’s also a lot of bed pressure as well. People are getting discharged fairly early from an inpatient rehab setting so they still have quite high care needs and so … physical assistance is required from carers in a lot of cases” (Participant 7).

For participants working in a community setting, they discussed the challenge of providing support to carers due to billable targets and waitlists. One participant reported “it becomes very money focused” (Participant 11). For another participant they reported “a lot of the time it circles back to well this funding is for person X not for their carer” (Participant 6).

Many participants working in both community and hospital settings discussed the challenge of policies and funding restrictions through agencies such as Australia’s National Disability Insurance Scheme (NDIS). Participants discussed the challenge of often spending additional time to advocate for stroke survivors and carers and not receiving what they requested.

“From an NDIS [National Disability Insurance Scheme] perspective it’s very time consuming, challenging, we’re often not getting what we’re recommending in our planning. What we’re recommending isn’t always getting approved through the planning meetings. So it’s often a get home see if they fail, and then they might try and put in more supports” (Participant 7).

Some participants discussed how often carers are not included or considered in funding packages which placed additional stress on the healthcare professional.

“But we’re like, going against what they’ve [National Disability Insurance Scheme] said, and we’re doing this, it becomes very overwhelming for us and for people. And you know, a lot of time, we might not have the funding to do as much as we want, or we might not have the funding” (Participant 6).

Other participants reported that lack of flexibility of funding bodies which impacted on changes to therapy schedules and resulted in additional work. Another challenge reported by participants was the prolonged wait for the stroke survivor to receive NDIS funding approval. Participants discussed how often carers would have to provide additional support due to the lack of access to funding for equipment and paid supports.

“That prolonged wait to get decent proper supports and that’s probably a big issue because the carers pick up a lot of that caring in the initial phases. I’ve got a guy going home next week who still doesn’t have his NDIS [National Disability Insurance Scheme] access and it’s just going to be him and his wife and he has three flights of stairs like come on, that is a big ask for someone…However, they’re not going to have a lot of support until their NDIS kicks in and I think that’s really unfortunate” (Participant 8).

*Priorities in practice*. There was a consensus among participants that providing informal carers with support is a shared responsibility. The majority of participants felt that all healthcare professionals have a role to play in identifying when a carer requires support and providing carer training and education. One participant reflected on the complexities of supporting carers and the need to target multiple areas when providing support.

“I think everyone has a part to play because we are so involved with the patient, we know what’s going on with the patient. There is so much to carer support… That one aspect of needing counselling, needing to know what services and resources they can tap into. Giving them [carer] that knowledge and understanding of what’s going on in that patient’s journey” (Participant 5).

Other healthcare professionals felt that whilst supporting the health and wellbeing of carers should be a shared responsibility, it often (by default) becomes the role of the social worker or occupational therapist. One participant stated “I would like to think that it would be all of us. I think it’s probably predominantly the social worker because they are really involved in that psychosocial support” (Participant 10).

Whereas other participants felt that supporting carers was not really the primary focus of their role.

“My mentors, they were very much, you know, how do we give as a challenging exercise as possible? How do we get as much intensity practice? Involving the carers was seen as a pathway to get more practice” (Participant 2).

A major priority that was frequently discussed by participants was the need to provide as much structured therapy to the stroke survivor as possible. These participants discussed how through prioritising therapy for the stroke survivor, they did not have sufficient time to provide support to the carer. Supporting carers was described as a “peripheral on the radar” and “secondary to the benefits for the patient”.

“I know sometimes when we go through our day to day, it can be very busy and I guess normally our priority as a clinician is providing that therapy for the patient” (Participant 5).

### Recommendations for a program designed for carers

Based on their experiences of working in stroke care, participants discussed 2 major areas that must be considered when supporting carers. These themes were: (1) Content and information delivery; and (2) Timing of support.

#### Content and information delivery

The majority of participants discussed the importance of needing additional resources that are tailored to the needs and priorities of carers. When participants were asked what would be important to include in such resources, they reported a variety of topics including stroke education, information on support services, carer fatigue and stress, and how carers can look after themselves. Participants identified the importance of combining this information into a booklet or package to ensure it is accessible and useful for carers.

“I’m picturing a booklet or something I’m picturing somewhere where they can write stuff down… I think that would be a cool thing if it’s got some information and where it’s got some space where that carer can write their own information” (Participant 8).

Many participants felt that providing carers with information on stroke education was extremely important to ensure they felt confident and prepared to take on the caring role. Participants discussed including information on common symptoms after a stroke, the recovery process of a stroke, how to prevent a secondary stroke, and information on manual handling for assisting with transfers.

“Some sort of video modules on common things like common symptoms after a stroke…like education on manual handling, education on things like common symptoms after a stroke, such as neglect, and how we can support people with neglect, like education on, you know, how to best have someone seated up for a meal” (Participant 1).

Other participants identified the importance of including information on where carers could access existing support services such as helplines, carer organisations, and carer support groups.

“A couple of things is a phone number that you can call … like you know, lifeline or Beyond Blue … phone call or chat type services. So that if someone is really having a bad day and then we don’t want them taking it out on that patient we want them to phone someone and get some help straight away” (Participant 8).

Participants also reflected on the importance of including specific information on carer fatigue and stress to ensure carers could readily recognise these signs. These participants also identified the importance of including information around how a carer can look after themselves such as ensuring they maintain their physical health through eating regular meals and exercising.

“What is carer burnout? What is it that they are experiencing? What is fatigue? Why does this happen? How often it happens? Something that would be easy to digest and simplified around that. Just as like a context to start off with because we have come across people who are experiencing it yet but they’ve never actually heard these terms before. They don’t realise like what it is, they just feel tired and sad and things like that” (Participant 6).

“I think in terms of what could be included in a program…I mentioned the hobbies and what they could do at home but maybe things like healthy eating…the carers not eating because they’re too worried about making the consistency food for their loved one, or their loved one wasn’t eating, so then they weren’t eating, the importance that that food has on your own energy, and your ability to actually care for someone and their need to have medical checkups because they’re constantly in the doctor’s office for their loved ones, they actually weren’t going out and looking after their own health and ailments at the time” (Participant 9).

A few participants identified the importance of providing information to carers around taking rest breaks and having time to engage in their own activities of interest. This was discussed as important to reduce carer burnout.

“I think some of that question might also come from an understanding about not just carer burnout but just general health and wellbeing, so you know, even things like exercise or hobbies. So I remember saying previously to a client’s carer, you know, are there any hobbies that you can do to distract you from let’s just say the, I hate to say, burden, but maybe the burnout that they were experiencing at the time. So that they didn’t feel that all of their thoughts and time was focused on the individual that they also needed to think about themselves and if they’re not healthy, how can they care for someone else?” (Participant 9).

Participants discussed the importance of delivering information in multiple ways to ensure it was suitable to the carers’ preferences and learning styles. Suggestions included using multimodal resources such as videos and a booklet, providing resources in languages other than English, and ensuring resources are suitable for carers with lower levels of health literacy.

“Having different kinds of resources for different people that learn different ways… Maybe just tailoring it to different people and how they differ and how they learn differently. And taking into account that we might have a large CALD [Culturally and Linguistically Diverse] and non-English speaking background as well and tailoring it to maybe the lower health literacy rates” (Participant 3).

Participants also identified the importance of considering the time required for carers to engage in a program or read through resources, suggesting that any resources provided to carers should be a “quick guide” and not time consuming to allow the carer to spend more time with the stroke survivor or completing their daily activities.

“Probably not super time intensive like it needs to be like a quick guide … there’s so much information and so much that they’re having to do when they’re having to spend time with their loved one, and they’re having to then still go home and eat and shower” (Participant 10).

#### Timing of support

When asked when carers should be provided with resources and support, most participants advised this should be provided during the ‘transition home phase’ or rehabilitation setting. Participants felt that providing support during the early stages of the caring role would ensure carers are aware of the services and support available. Providing these resources and support earlier was also identified as key to reducing the risk of carer burden.

“It [support] should be done in the in the acute setting…what I have found is even some clients that don’t go to rehab that leave after the acute setting and go home, they still end up with long term support and by then potentially the clients been home for months before an OT [Occupational Therapist] comes on board” (Participant 9).

Other participants discussed how it was important to not provide carers with too many resources initially as it could be counterproductive and result in overwhelming the carer rather than informing them.

“If you do it [provide resources] too early it’s very easy to overwhelm that person and can actually mean that they may be a bit more reluctant to actually be the carer because they’ve suddenly taken on all of this information very early and they’re just not ready for it” (Participant 8).

One participant reflected on the importance of ensuring that carers were provided with sufficient time to review resources but also have a point of contact if they required assistance.

“I think a tricky thing would be timing … I think what’s probably important is being able to do it [resource] as a self-paced but knowing that there’s someone that they can email or text or call, if they need you know assistance” (Participant 10).

### Future priorities in stroke care

Based on their experiences, participants reflected on what changes need to be made in stroke care to better support carers. Two themes were identified to capture these views: (1) Empowering health care professionals; and (2) Less judgement and more structure.

#### Empowering health care professionals

Many participants discussed the need for additional support and resources to be able to provide carers with training and psychosocial support. Participants identified the need for a shift in the view of carers, formalised training, continuing professional development workshops, and useful resources.

Participants discussed the lack of recognition, within stroke care, of the role of a carer and how life changing adopting this role can be. Many participants reported there needed to be a shift in practice to involve carers and provide additional support.

“I guess there’s not as much recognition on how life changing a stroke can be for a carer… obviously there is a lot of recognition on how life changing it can be for a stroke survivor” (Participant 1).

Participants reflected on the importance of formalised training, workshops, and other resources to increase carer recognition and equip healthcare professionals to better provide support specifically to carers. Many participants reported that they were often not aware of resources or training available and therefore increasing recognition of this would help healthcare professionals to support carers.

“I think making that awareness and having that formal training, I guess more available, more spoken about would definitely be something that will be beneficial and will make us I guess, a bit more aware as clinicians as well” (Participant 5).

Many participants identified the importance of having access to carer resources to improve outcomes such as carer stress and burden. These participants reflected on how these resources would reduce the burden on healthcare professionals by reducing time spent on creating these resources.

“Having some resources there would be really helpful because I think it is an area that is sort of missed a bit like we touch on it, but it’s not the biggest priority and spending the time on it [resource development] can be challenging” (Participant 7).

Other participants, while discussing already having access to several resources, saw a need for training to equip them on how to best support the carer.

“I think more workshops and training …I think there’s brochures everywhere on things, and it just feels like sometimes even when I’m giving out the ‘my stroke journey’ packs it just feels like I’m going, here, your life’s changing, here’s lots and lots of information to read. Those resources do exist, and I have access to those resources. But I think more practical training would be the best thing” (Participant 1).

When asked about what they would like to see included in professional training workshops, health care professionals identified several topics including identification of early signs of carer stress and burnout, how to provide carer training, importance of involving the carer, and resources available for carers.

“Some feedback around the fact that [supporting carers] is important like, this is how carers are struggling. Some training to go with that just to say, this is what you can actually do… and resources for supporting carers” (Participant 1).

“From a CPD [continual professional development] perspective it would be beneficial to have some kind of identification of carer burnout and training” (Participant 9).

Some participants felt having access to training and resources would improve their confidence and outcomes for carers and stroke survivors.

“Handouts probably I think I what I like in terms of resources for anything, is some training for the therapist and the handout that goes with that. So I guess it kind of helps us to educate around what we’re actually giving … If I had an hour and a half of education on this handout, I would feel pretty confident to talk to it” (Participant 4).

#### Less judgment, more structure

Participants felt that healthcare professionals should ensure they involve and check in on carers more frequently. Many participants however, discussed that one of the main barriers to involving and supporting carers in practice was relying on clinical indication or the motivation of the healthcare professional. This was seen as problematic by many participants who felt that the involvement of carers should not be based on the initiative of healthcare professionals, but rather be embedded within their process.

“It [carer training] shouldn’t be really up to personal preference, I think it’s like a really important skill that should be able to be transferable and be a bit more objective. And I think, again, like yes, working on supporting carers and that, like, social and emotional side of things” (Participant 10).

“We would do carer training very frequently, but it’s like if it was clinically indicated, and they, the therapist thought about it, then that will do it. But it’s not something that’s like a tick box to discharge the patient if that makes sense” (Participant 2).

A few participants reflected on how supporting carers was often not identified as an important skill to develop. Therefore, many participants felt that healthcare professionals do not spend time on this development. One participant reported that it was often “expected” that as healthcare professionals they knew how to support stroke survivors and they also had the skills to teach carers.

“I probably never really thought that it [professional development and training] would be a thing because I’ve never even seen it… Now that we talk about the fact that it is very much just based on my personal experience, well, why should it be if it’s a skill that a clinician needs to have. So I think that is something that would be a barrier that it’s probably not seen as a skill to be developed and it’s probably more seen as an expectation of something that you just do… it’s kind of just assumed that because you know how to do it, you’ll be able to know how to teach a carer” (Participant 10).

A few participants suggested the need for a more objective and standardised approach to supporting carers within stroke care. When asked what was feasible to implement within the stroke care setting, participants advised the inclusion of prompts or a standardised checklist that would ensure that healthcare professionals have considered the need for carer training and support. Participants reported that the use of an assessment, “prompt” or “checklist” may also assist healthcare professionals in screening for carer stress and burden. Some participants felt that the inclusion of a prompt would ensure checking on carers and that providing support was embedded within their routine practice.

“I guess you could also have like a prompt somehow or have some kind of requirement is carer training indicated yes or no? If yes, have we done it? I hope we do that without needing to be asked” (Participant 2).

“I wonder if maybe something like an automatic prompt……But we don’t really address carer specifically, I wonder if maybe like, a neurological carer needs checklist or something like that might be something interesting that we can implement-. But it might also be something useful in the acute setting, to screen for like psychological stress, if there was carer burden” (Participant 3).

One participant advised the need for a standardised education approach that provided carers with an opportunity to attend weekly sessions for information and ask any questions that might assist them in the caring process.

“There isn’t much time to check in with those carers and have a conversation and provide education. It is more done close to discharge, but potentially having more check ins early on if we know they’re going to be the carer would be better and maybe even having like an allocated discussion time where we say well this is a half an hour for this week, If you want to ask questions or you feel like you need some information … open up those communication channels and make it a little bit more inclusive with the carers I guess as well” (Participant 8).

## Discussion

To our knowledge, this is the first study to explore the experiences and perceptions of healthcare professionals working in stroke care on how to best support informal carers and provide recommendations for practice and a carer support program. Three overarching categories were used to organise identified themes: (1) Experiences of working in stroke care and supporting carers (3 themes, 4 subthemes); (2) Recommendations for a program designed for carers (2 themes); and (3) Future priorities in stroke care (2 themes).

The category of experiences of working in stroke care and supporting carers included themes of how healthcare professionals support carers, skills and knowledge of healthcare professionals, and priorities and pressures of clinical practice. Participants acknowledged the important role of healthcare professionals in supporting carers and were able to reflect on the different strategies they utilise to support carers, both practically and emotionally. Participants discussed strategies such as identifying a ‘key worker’ as the main point of contact for carers and use of collaboration between healthcare professionals. Another strategy was the use of a ‘trial of care’ period to help carers feel more prepared and know what to expect in their role. A particular finding of note in the current study of healthcare professionals in Australia is that they were able to use these strategies to provide carers with opportunities to identify the extent of their involvement in providing care. This is contrary to the findings of Ang et al. [12], who found that healthcare professionals in Singapore were unaware of the varying expectations regarding extent of carer involvement.

Participants identified various challenges and priorities which impacted on their ability to support carers. One of the main priorities discussed within stroke care was the need to provide as much therapy to the stroke survivor as possible. Therefore, supporting carers was identified as more of a peripheral focus to healthcare professionals. Participants reflected on additional challenges such as usual work hours, understaffing, lack of resources, pressure to discharge patients, and funding bodies. These findings support those of Connor et al. [33] that found healthcare professionals can experience barriers to supporting carers such as staff shortages and inadequate support packages.

The second category was recommendations for a program designed for carers. The themes identified within this category included content and information delivery and timing of support. Participants acknowledged the need for resources and training that are tailored to the specific needs and priorities of carers, and that are not time consuming. Participants identified the importance of designing resources that were suitable for culturally and linguistically diverse carers and delivered through multiple formats (i.e., video, booklets). Participants reflected on the importance of educating carers on a variety of topics including stroke education, information on support services, carer stress and fatigue, and how to look after oneself. These findings are in line with previous studies that have examined the experiences of carers and indicated carers reported requiring more tailored and specific information as opposed to brochures and pamphlets [14, 34]. The timing of support and resources was discussed by participants as an important consideration when designing a program for carers. Participants identified the importance of providing carers with support through training and resources early in the rehabilitation process or acute setting. Participants reiterated the importance of ensuring carers were not overwhelmed with too many resources. This finding supports those of Walker et al. [35], that reflected the experiences of carers who reported that the optimal timing of resources or intervention is early in the stroke pathway.

The final category of future priorities in stroke care included two identified themes of (1) empowering healthcare professionals, and (2) less judgement, more structure. Healthcare professionals identified that there was often a lack of recognition of the role of a carer and the importance of supporting the carer. This finding is in line with previous research highlighting that carers would like healthcare providers to be more empathetic and understanding of their role [36]. Healthcare professionals within the current study identified the need for further professional training and resources to raise awareness on the importance of carers. Interestingly, within this study many participants reported not receiving adequate formal training on how to support and train carers. The lack of training meant that healthcare professionals had some difficulty with identifying the early signs of carer stress and burden, and often lacked confidence to emotionally support the carer. Training of healthcare professionals and resource provision has been identified as an important approach that health systems can use to directly support carers [37].

A unique finding within this study was that healthcare professionals identified they would often rely on clinical indication or the personal initiative of a healthcare professional on whether carer training and support was required. Participants suggested the need for a more objective and standardised approach to supporting carers such as an assessment, checklist, or prompts to ensure they had considered the need for carer training and as a method to screen for caregiver burden. It is evident that screening for and addressing caregiver burden may improve physical health and psychiatric outcomes for carers [38]. A study by Riffin et al. [39] examining the needs of family carers of older adult patients identified a high degree of variability in assessments and approaches used to identify carer needs and outcomes. Riffin et al. [39] suggested a need for standardised carer assessments that are brief to administer, as well as practice guidelines to prepare clinicians for effective engagement of carers including assessment of carer needs and outcomes.

### Strengths and limitations

The present study provides a unique insight into healthcare professionals’ perceptions of supporting informal carers within stroke care and the challenges that are experienced. A particular strength of this study were the strategies (i.e., data triangulation, reflexivity, member checking) utilised to improve the credibility of data. Some potential limitations should be considered when interpreting these findings. First, the sample of 11 healthcare professionals was specific to Australian services and therefore may not be transferable to other healthcare settings with different models of care and funding. Second, the use of a purposive sampling strategy can introduce possible researcher bias. To alleviate the possibility of researcher bias, however, a clear selection criterion was utilised to screen participants for eligibility within this study. Third, while the perceptions of carers themselves were not included in this particular paper, they are important in order to design programs to enhance support, and as such will be the focus of a future study.

### Recommendations

The findings of this study suggest that there is a need for additional formal training and resources to equip healthcare professionals to better recognise and support carers within stroke care. Training of healthcare professionals and adequate resource provision can help to address challenges identified within stroke care. Further research is required to determine whether brief and standardised carer assessments can help to improve carer engagement and outcomes in stroke care. The implementation of standardised assessment tools may also allow for identification of carer needs and outcomes early in the stroke pathway.

Findings from this study highlight the importance of designing resources and implementing support around the needs and priorities of carers. Healthcare professionals should consider the timing of support and resources and ensure they are not overwhelming carers during the initial stage of their caring role. Future research should focus on the use of co-design methods to inform the development of programs and resources designed for carers.

## Supporting information

S1 Appendix Interview topic guide. (DOCX)

S2 Appendix Main themes with supporting extracts. (DOCX)
