# Supplementary material for: Healthcare professionals’ perceptions on providing support to informal carers within stroke care
Source: PLoS One. 2024 Oct 15;19(10):e0311915. doi: 10.1371/journal.pone.0311915 (PMC11478868; doi:10.1371/journal.pone.0311915)
Supplement: S1 Appendix — (DOCX) [file pone.0311915.s001.docx]

**S1 Appendix. Interview topic guide**

- Can you tell me a little about your current role and workplace?
- In your workplace are there any support or resources in place for informal carers of stroke survivors? If so, could you describe these?
- In what ways do you involve the carer in your current workplace?
- Can you tell me a little bit about the type of training, CPD, or skills set/development have you received to support informal carers of stroke survivors?
- Can you think of a time where you have provided support to an informal carer of a stroke survivor and tell me about this?
- Can you think of a time where you experienced a challenge when providing support to an informal carer, and tell me about that?
- What do you think is realistic for you to do as a health professional to support carers in your current workplace?
- In your current role, do you collaborate with other health care professionals to support for informal carers, and can you describe examples of what this looks like?
- What resources and support do you think is important to provide informal stroke carers?
- How do you think the current system can be improved to better meet the needs of stroke carers
- Based on your experience what do you think should be included in a program to support the health and wellbeing of carers?
- When do you think this program should be implemented during the stroke recovery and why?
