# Supplementary material for: Healthcare professionals’ perceptions on providing support to informal carers within stroke care
Source: PLoS One. 2024 Oct 15;19(10):e0311915. doi: 10.1371/journal.pone.0311915 (PMC11478868; doi:10.1371/journal.pone.0311915)
Supplement: S2 Appendix — (DOCX) [file pone.0311915.s002.docx]

| **S2 Appendix. Main themes with supporting extracts.** | | | |
| --- | --- | --- | --- |
| **Category** | **Theme** | **Sub-themes** | **Example quotes** |
| Experiences of working in stroke care and supporting carers | How healthcare professionals support carers | Key strategies from the field | “We often have a key worker that works with them [carer] and we often pick the person who they are spending the most time with or that they already have quite a good rapport with…I think if it's a multidisciplinary team approach and then it doesn't necessarily sit with one individual” (Participant 7). |
|  |  | Collaboration between healthcare professionals | “Right from the start, we always do things together and we also share the assessment bits as well. So the physiotherapist feels confident to do most of the OT [Occupational Therapist] bits and the OT can do most of the physiotherapy bits as well … even if one person is note taking throughout, but one person's giving advice and education to the actual carer as well, while the other person is doing the assessment with the patient. It's very collaborative in that front” (Participant 3). |
|  | Skills and knowledge of healthcare professionals |  | I can't think of anything specific in terms of CPD [continuing professional development]. No, I can’t identify a specific course that was directed towards carers or involving carers” (Participant 9) |
|  | Clinical practice: Priorities and pressures | Challenges in the workplace | “Often will give like the handouts and booklets for places like Carer Gateway and My Aged Care if they're older participants but yeah those are mostly the standard ones. We don't have too much specific out there for them [carer]” (Participant 7) |
|  |  | Priorities in practice | “The nature of just how sort of chaotic things get in that clinical environment, we're always prioritising how much time we spend in therapy with patients. I would say we don't often get to the phone as much as we should like to reach out and contact people that can take a good hour or more of your day” (Participant 2).  “We're so caught up in managing the patient and making sure that they're well supported from a mental and physical perspective, that oftentimes we're not we don't necessarily overlook, but we tend to prioritise the patient's problems over their carers because they're the person that we're treating, whilst also acknowledging carers too” (Participant 3). |
| Recommendations for a program designed for carers | Content and information delivery |  | “I think just the knowledge that there are things out there, like especially psychology and support groups and social groups, and that sort of stuff is out there. I think more knowledge for some people, some people don't realise what carer fatigue and carer stress is even if they're in it, they don't realise how common it is or what it is, it's just like, this is my life now” (Participant 6).  “I think really a good understanding of just taking breaks … I'm sort of making that clear about, you know, you're sort of making this like comparing it to a paid work situation, what it would actually look like, so that they have an idea about how much work it can actually be. Because I think then sometimes people will be more open to a bit more help initially if they could sort of picture in their minds what it might actually look like you know a few weeks down the track” (Participant 7). |
|  | Timing of support |  | “If you are calling it a carers program I would say towards the later half of the inpatient stay, because then we're hopefully having a bit more knowledge around what the patient might need. And then when you'd be I guess labelling that like support for you [carer]” (Participant 2). |
| Future priorities in stroke care | Empowering health care professionals |  | “It's about awareness that those things [resources] even exist and if clinicians don't know that they exist then how are they going to share that with clients or carers?” (Participant 10)  “Increasing awareness of the fact that there certainly is carer burden out there. Sometimes we don't necessarily think about it or prioritise it and then as a result, people slip through the cracks, there's worse outcomes down the track, there's sub optimal care or there's just burn out very quickly, So maybe just making people more aware of that and the resources out there that we can actually access in regards to trying to minimise the carer burden… if there's any specific training, or principles, or there's any models or anything like that, that we could maybe structure ourselves around” (Participant 3). |
|  | Less judgement, more structure |  | “Having something embedded in your routine to make sure that you have engaged carers” (Participant 2). |
